# Immunohistochemical and Morphometric Analysis of Lung Tissue in Fatal COVID-19

**DOI:** 10.3390/diagnostics14090914

**Published:** 2024-04-27

**Authors:** Ioana-Andreea Gheban-Roșca, Bogdan-Alexandru Gheban, Bogdan Pop, Daniela-Cristina Mironescu, Vasile Costel Siserman, Elena Mihaela Jianu, Tudor Drugan, Sorana D. Bolboacă

**Affiliations:** 1 Department of Medical Informatics and Biostatistics, Iuliu Hațieganu University of Medicine and Pharmacy, 400349 Cluj-Napoca, Romania; andreea.gheban-rosca@umfcluj.ro (I.-A.G.-R.); tdrugan@umfcluj.ro (T.D.); 2 Clinical Hospital for Infectious Diseases, 400348 Cluj-Napoca, Romania; 3 County Emergency Clinical Hospital, 400006 Cluj-Napoca, Romania; 4 Department of Histology, Iuliu Hațieganu University of Medicine and Pharmacy, 400349 Cluj-Napoca, Romania; marina.elena@umfcluj.ro; 5 The Oncology Institute “Prof. Dr. Ion Chiricuță”, 400015 Cluj-Napoca, Romania; bogdan.pop@umfcluj.ro; 6 Department of Anatomic Pathology, Iuliu Hațieganu University of Medicine and Pharmacy, 400347 Cluj-Napoca, Romania; 7 Forensic Institute, 400006 Cluj-Napoca, Romania; dana_pvl@yahoo.com (D.-C.M.); csiserman@umfcluj.ro (V.C.S.); 8 Department of Forensic Medicine, Iuliu Hațieganu University of Medicine and Pharmacy, 400006 Cluj-Napoca, Romania

**Keywords:** severe acute respiratory syndrome coronavirus 2 (SARS-CoV-2), immunohistochemistry, lung pathology, pneumocytes, macrophages

## Abstract

The primary targets of severe acute respiratory syndrome coronavirus 2 (SARS-CoV-2) in the lungs are type I pneumocytes, macrophages, and endothelial cells. We aimed to identify lung cells targeted by SARS-CoV-2 using viral nucleocapsid protein staining and morphometric features on patients with fatal COVID-19. We conducted a retrospective analysis of fifty-one autopsy cases of individuals who tested positive for SARS-CoV-2. Demographic and clinical information were collected from forensic reports, and lung tissue was examined for microscopic lesions and the presence of specific cell types. Half of the evaluated cohort were older than 71 years, and the majority were male (74.5%). In total, 24 patients presented diffuse alveolar damage (DAD), and 50.9% had comorbidities (56.9% obesity, 33.3% hypertension, 15.7% diabetes mellitus). Immunohistochemical analysis showed a similar pattern of infected macrophages, infected type I pneumocytes, and endothelial cells, regardless of the presence of DAD (*p* > 0.5). The immunohistochemical reactivity score (IRS) was predominantly moderate but without significant differences between patients with and without DAD (*p* = 0.633 IRS for type I pneumocytes, *p* = 0.773 IRS for macrophage, and *p* = 0.737 for IRS endothelium). The nucleus/cytoplasm ratio shows lower values in patients with DAD (median: 0.29 vs. 0.35), but the difference only reaches a tendency for statistical significance (*p* = 0.083). Our study confirms the presence of infected macrophages, type I pneumocytes, and endothelial cells with a similar pattern in patients with and without diffuse alveolar damage.

## 1. Introduction

The emergence of the novel coronavirus—severe-acute-respiratory-syndrome-coronavirus 2 (SARS-CoV-2)—in late 2019 started a three-year-long global pandemic with an impact on health systems, communities, and economies [1]. The SARS-CoV-2 belongs to the *Coronaviridae* family and is classified under the *Betacoronavirus* genus [2]. These viruses are enveloped, single-stranded, positive-sense ribonucleic acid (RNA) viruses with a distinctive crown-like appearance under electron microscopy (EM). The viral genome encodes essential structural and non-structural proteins, each playing a role in the viral lifecycle [3]. Among these proteins, the nucleocapsid (N) protein is a component in the viral assembly and propagation [3]. The N protein, approximately 500 amino acids long, is the most abundant protein within the virion, occupying the core and constituting 25–30% of its total mass. This multifunctional protein forms a ribonucleoprotein complex with the viral genome, essential for RNA packaging, nucleocapsid formation, and virion assembly [4]. Additionally, the N protein interacts with various host factors, modulating viral replication and pathogenesis. Its role makes it a promising target for antiviral treatment development and diagnostics [5].

The primary route of SARS-CoV-2 infection involves the respiratory tract. The virus attaches to host cells in the upper and lower respiratory tract through its spike (S) protein, interacting with the angiotensin-converting enzyme 2 (ACE2) receptor [6]. Following membrane fusion and viral entry, the viral genome is released into the cytoplasm, initiating replication and protein synthesis [7]. The newly synthesized N protein binds to the viral RNA, forming nucleocapsids in the cytoplasm. These nucleocapsids assemble with other structural proteins in the endoplasmic reticulum and Golgi apparatus, culminating in the formation of new virions that are finally released from the infected cell, perpetuating the infection cycle [8]. Despite its initial infection sites, the most severe clinical manifestations of coronavirus disease 19 (COVID-19) emerge from damage to the lower respiratory tract, particularly the lungs. Viral infiltration and replication within the lung cells, primarily alveolar epithelial cells and pneumocytes, trigger an inflammatory cascade leading to pneumonia, acute respiratory distress syndrome (ARDS), and other complications [9]. A massive release of inflammatory molecules by immune cells, known as a “cytokine storm”, is considered to be a major culprit behind ARDS, multiple organ failure, and even death in COVID-19 patients [10]. This storm involves various immune cells, like B cells, T cells, and macrophages, all releasing high levels of pro-inflammatory cytokines such as interleukin-1 (IL-1), IL-6, and tumor necrosis factor-α (TNF-α), linked to the severe lung damage observed in many COVID-19 cases [11].

**Figure 1:**
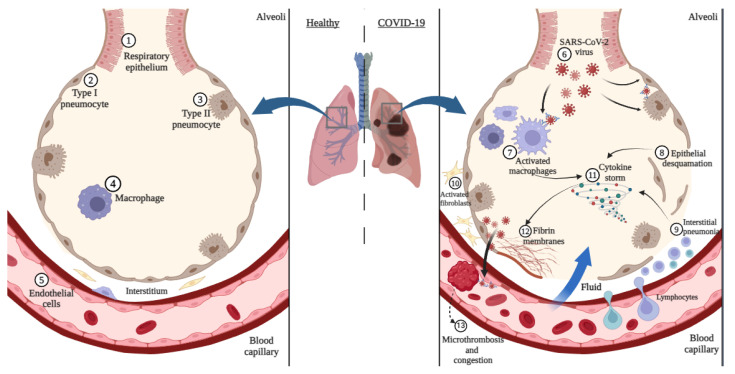
Schematic representation of normal histology and the pathogenesis of COVID-19 at the alveolar level (created with BioRender on 2 March 2024).

The alveolar epithelium—the air-blood barrier in the lungs (Figure 1)—comprises type I and II pneumocytes resting on a capillary network lined by endothelial cells and a variable amount of alveolar and interstitial macrophages [12]. Within the alveolar epithelium, two specialized cell types, type I and type II pneumocytes, ensure efficient gas exchange and maintain lung structure. Type I pneumocytes, covering approximately 2/3 of the alveolar surface area, are thin and squamous, resembling pavement cells [13].

Type II pneumocytes are cuboidal in shape and possess more prominent organelles and microvilli. Their primary role is the production of surfactant, a lipoprotein complex that reduces surface tension within the alveoli, preventing their collapse during exhalation [14]. Additionally, they act as progenitor cells, dividing and differentiating into type I pneumocytes to replenish damaged ones, ensuring lung repair and regeneration, and creating fibrosis in pathologic conditions [15]. While type II pneumocytes share a similar morphology to macrophages, with a similar nucleus-to-cytoplasm ratio, they are distinct from macrophages, as they support the immune system by producing specific signaling molecules and interacting with immune cells [16]. Macrophages found both within the alveoli and the interstitial space are a type of white blood cell derived from monocytes and are primarily responsible for phagocytosis and antigen presentation, contributing to the immune defense system within the lung [17].

SARS-CoV-2 infection damages the alveolar structure, leading to a spectrum of histopathological changes documented in autopsy studies (Figure 1). The most prominent finding is diffuse alveolar damage (DAD), characterized by exudative, organizing, and fibrotic phases [18]. During the exudative phase, alveolar spaces fill with protein-rich fluid (edema), desquamated pneumocytes, and inflammatory infiltrates composed of lymphocytes, macrophages, and neutrophils. This disrupts the air-blood barrier and impedes gas exchange [19]. The organizing phase is marked by the proliferation of fibroblasts and the deposition of fibrin, leading to partial airspace obliteration. In severe cases, the fibrotic phase ensues, characterized by excessive collagen deposition and parenchymal scarring, potentially leading to long-term respiratory compromise [20]. Additionally, microthrombi formation within the alveolar capillaries further contributes to hypoxemia by obstructing blood flow and leading to intra-alveolar hemorrhages and red infarctions of various degrees of severity, as all caliber of vessels are affected due to endothelial dysfunction induced by the virus [21]. In contrast to *herpes simplex* (HSV), *rubella* virus, *cytomegalovirus* (CMV), and *respiratory syncytial virus* (RSV), SARS-CoV-2 does not typically form distinctive, easily identifiable, histological inclusions in lung tissue. Most viral-related changes include hyperplasia, hyperchromasia, enlarged and irregular nuclei, metaplasia, or multinucleation [22].

Evidence of the presence and extent of SARS-CoV-2 infection in lung tissue is vital for diagnosis, research and assessing therapeutic interventions. Researchers currently use various methods, each with its advantages and limitations, to accurately detect the presence and extent of SARS-CoV-2 infection in lung tissue [23]. One widely used approach is a real-time reverse-transcription polymerase chain reaction (RT-PCR), a highly sensitive technique able to detect viral RNA and indicate active infection. However, a RT-PCR often requires specialized equipment and technical expertise, limiting its accessibility in certain settings [24]. Immunohistochemistry (IHC) offers an alternative by targeting the N protein itself. This technique utilizes specific antibodies to visualize infected cells within tissues, providing insights into the spatial distribution and extent of viral infiltration. While slightly less sensitive than RT-PCR, IHC is valuable for its ability to provide morphological context and correlate viral presence with tissue damage [25]. Another emerging technique is in situ hybridization (ISH), which identifies viral RNA directly within tissues. The ISH technique offers advantages over RT-PCR by preserving spatial information and enabling the visualization of infected cells [26]. Finally, EM provides the highest-resolution view of viral particles within tissues, allowing for the direct visualization of virions and their interactions with host cells [27].

In this study, we aimed to identify and characterize the lung cells infected by SARS-CoV-2 and to determine their distribution within the lungs using immunohistochemical staining for the viral nucleocapsid protein. The findings could provide insights into the role of the virus in the development and progression of the disease.

## 2. Materials and Methods

The investigators ensured adherence to the ethical principles stipulated in the Declaration of Helsinki during all stages of the study. The Iuliu Hațieganu University of Medicine and Pharmacy Cluj-Napoca Ethics Committee (DEP67/14 December 2021) and the Institute of Legal Medicine, Cluj-Napoca (2406/XII/703/24 March 2022) reviewed and approved our research protocol.

### 2.1. Study Design

**Figure 2:**
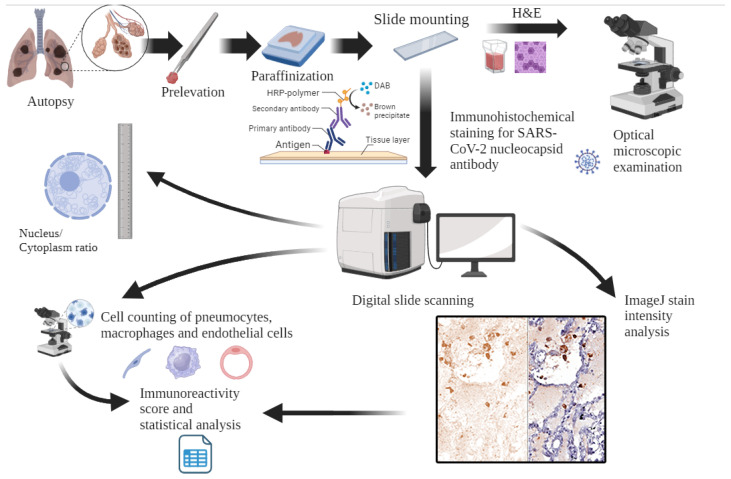
Workflow of histology slide preparation, microscopic examination, and image-based analysis (created with BioRender on 2 March 2024).

We conducted an observational cohort study at the Institute of Legal Medicine Cluj-Napoca, Romania, encompassing all autopsies performed by forensic pathologists between April and December 2020. We included in our study deceased individuals who tested positive for SARS-CoV-2 ante- or post-mortem, underwent complete autopsy during the study timeframe, and had both lung fragments harvested (Figure 2). To ensure data quality, we excluded cases where tissue formaldehyde fixation was insufficient. We retrospectively collected demographics and clinical data of evaluated patients from forensic reports.

### 2.2. Tissue Processing and Immunohistochemistry Staining

Lung tissue specimens underwent routine fixation in 10% neutral buffered formalin for 24 h, incorporating an antigen retrieval buffer. Thin sections (3 µm) were prepared from the paraffin blocks and subjected to dual staining. One stain employed Hematoxylin-Eosin for general tissue visualization, while the other utilized recombinant anti-SARS-CoV-2 nucleocapsid protein antibody (EPR24334-118—Abcam, Cambridge, UK) (RRID: AB_2788968). The immunohistochemical protocol involved deparaffinization with xylene and sequential washes with alcohol and water. Antigen retrieval, peroxidase blocking, and unspecific protein blocking were performed, followed by incubation with primary antibodies against the SARS-CoV-2 nucleocapsid for one hour. Secondary antibody incubation, DAB development, and Hematoxylin counterstaining completed the staining process (Figure 2).

### 2.3. Microscopic Examination

Microscopic slides were digitally scanned at 40× magnification using the 3D HISTECH PANNORAMIC SCAN II (Budapest, Hungary). Morphometric analysis was subsequently performed with a 3D HISTECH software Slide-Viewer v2.7 (Budapest, Hungary) (Slide-Viewer, RRID: SCR_017654). To ensure consistency, all images were analyzed using identical parameters, scan settings, and hardware versions, with a slide pixel dimension of 112,640 × 243,200 and over 9000 scanned fields of view. We used infected placental tissue as an external control. Immunopositivity was evaluated by calculating the Optical Density of each cell type using ImageJ—Fiji v.154i (LOCI, University of Wisconsin, Madison, WI, USA) (ImageJ, RRID: SCR_003070) for color deconvolution and pixel measurements. Immunopositive pneumocytes, endothelial cells, and macrophages were counted. Histological architectural distortions and similar morphologies made it difficult to differentiate type II pneumocytes from macrophages. Therefore, these cells were counted together as macrophages (Figure 2). The nuclear/cytoplasm ratio for each cell type was calculated using the formula N/C = N2/(C2 − N2) after counting the cells on ten high-power fields in each case with nuclear and cytoplasmic measurements. Analysis of the IHC slides employed a standardized immunoreactive score (IRS) system for immunopositive pneumocytes, macrophages, and endothelial cells. This composite score incorporates both the percentage of positive cells (score A) and the intensity of staining (score B). The addition of these individual scores (A + B) generates the final IRS score, ranging from 0 to 7.

### 2.4. Statistical Analysis

Our hypothesis was that immunoreactivity is different in patients with and without DAD, so data are reported on the cohort, and comparisons are made between these two groups. Immunoreactivity scores on the IRS scale (0–7) were categorized as follows: 1–2 (low), 3–5 (moderate), and 6–7 (intense). Shapiro–Wilk test (*p* < 0.05) and Q-Q plots rejected the normality of quantitative data and, therefore, data are presented as medians and interquartile ranges (IQR). Categorical variables are reported as frequencies and percentages. Fisher’s exact or Chi-squared tests, depending on the expected frequency counts, evaluated associations in contingency tables. We utilized the non-parametric Mann–Whitney test for comparisons between groups with non-normally distributed continuous variables. All tests were two-sided with significance set at *p* < 0.05.

Simple Interactive Statistical Analysis (SISA by Quantitative Skills, Available Online: http://www.quantitativeskills.com/sisa/ (accessed on 14 February 2024)), Mann–Whitney U Test Calculator by Social Science Statistics (available online: https://www.socscistatistics.com/tests/mannwhitney/default2.aspx (accessed on 14 February 2024)), IBM SPSS trial version v26 (Armonk, NY, USA) (IBM SPSS Statistics, RRID: SCR_019096), and Microsoft Office Excel 365 (Redmond, WA, USA) (Microsoft Excel, RRID: SCR_016137) were used for statistical description and analysis.

## 3. Results

### 3.1. Cohort Characteristics

We evaluated fifty-one autopsy cases, with patients’ ages ranging from 34 to 96 years and a median age of 71 years (Table 1). All the patients were unvaccinated, and almost two-thirds (64.7%, *n* = 33) were from urban areas. Nearly half (49%, *n* = 25) died outside of a healthcare institution, and 50.9% (*n* = 26) had associated comorbidities. Among the reported comorbidities, cardiovascular diseases were the most frequently reported, including hypertension (33.3%, *n* = 17) and heart failure (11.1%, *n* = 3), followed by metabolic disorders like obesity (Table 1) and diabetes mellitus (15.7%, *n* = 8).

### 3.2. Microscopic Features

Most patients (Table 2) showed varying degrees of congestion upon microscopic examination of lung tissue. Specifically, 19.6% (*n* = 10) presented severe congestion, 31.4% (*n* = 16) moderate congestion, and 35.3% (*n* = 18) mild congestion. Slight epithelial desquamation was observed in approximately one-quarter (23.5%, *n* = 12) of the cases. The nuclei of macrophages appeared enlarged and irregular, while the cytoplasm exhibited vacuolation and structural alterations. The infected cells were found to be distributed heterogeneously in the lung fragments. While some areas showed a substantial increase in infected cells, other regions remained unaffected. Table 2 summarizes the observed microscopic features in the lungs.

### 3.3. Morphometry and Immunoreactivity

Immunohistochemical analysis revealed the presence of infected macrophages in all samples, regardless of the presence of DAD (Table 3). Most patients (70.6%, *n* = 36) had detectable infected type I pneumocytes, with 75% (*n* = 18) in the DAD group and 66.7% (*n* = 18) in the group without DAD (Chi-square test: χ^2^ = 0.4, *p*-value = 0.5145). Infected endothelial cells were detectable in 58.8% (*n* = 30) of patients, 66.7% (*n* = 16) in the DAD group, and 51.9% (*n* = 14) in the group without DAD (Chi-square test: χ^2^ = 1.2, *p*-value = 0.2883). The morphometric and immunoreactivity characteristics are summarized in Table 3.

**Figure 3:**
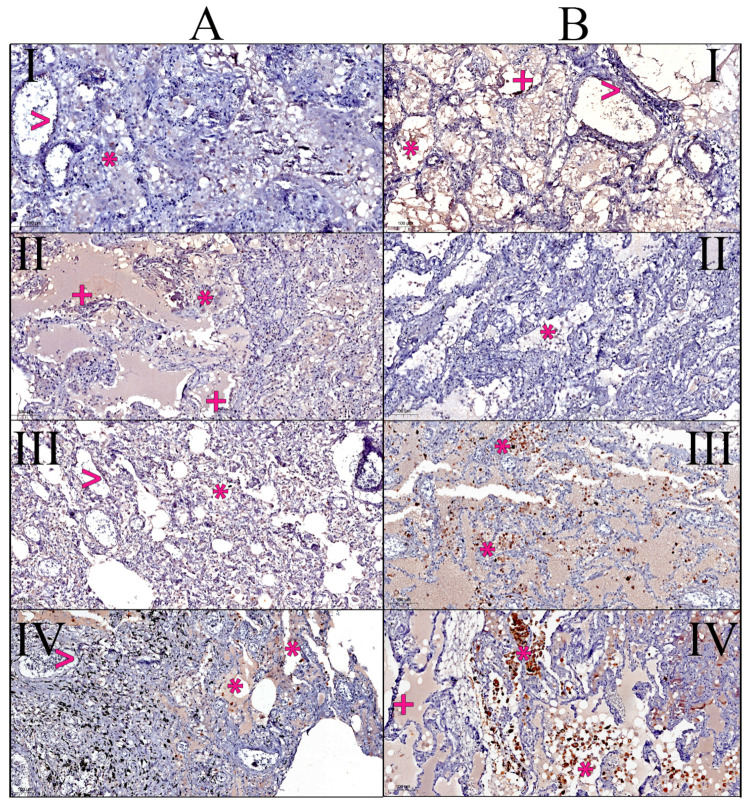
Immunohistochemical stain for SARS-CoV-2 nucleocapsid by example. Column (**A**) presents patients with DAD: **I**—53-year-old male presenting scarce immunopositive endothelial cells (>) and macrophages (*); **II**—67-year-old male with positive type I pneumocytes (+) and macrophages (*); **III**—87-year-old female with rare positive endothelial cells (>) and macrophages (*); **IV**—81-year-old male with positive endothelial cells (>) and numerous positive macrophages (*). Column (**B**) presents patients without DAD: **I**—52-year-old male presenting numerous positive endothelial cells (>), type I pneumocytes (+), and macrophages (*); **II**—67-year-old male with sparse immunopositive macrophages (*); **III**—88-year old female with highly immunoreactive macrophages and type II pneumocytes (*); **IV**—80-year-old male presenting abundant immunoreactive macrophages and type II pneumocytes (*), as well as type I pneumocytes (+). All images were taken at 20× magnification in the COVID-19 nucleocapsid IHC stain.

Figure 3 illustrates specific cases, showing the immunohistochemical stain for SARS-CoV-2 nucleocapsid on patients with (column A) and without (column B).

## 4. Discussion

Our study emphasizes the complex molecular interactions between SARS-CoV-2 and various lung cell types. While we have not pinpointed a specific cell type solely responsible for triggering DAD, we observed a high presence of infected macrophages with varying degrees of infection in type 1 pneumocytes and endothelial cells (Table 2). These observations may indicate a synergistic effect between all infected cell types in the onset of DAD, alongside a dysregulated immune response. No statistically significant differences in comorbidities, age, or sex were associated with the presence or absence of DAD. Furthermore, we noted that most patients with DAD had interstitial pneumonia (72.5%), which was previously reported on hematoxylin and eosin staining [28].

### 4.1. The Role of Evaluated Cell Type in COVID-19 Lung Disease

Type I pneumocytes facilitate gas exchange but are susceptible to viral entry and damage, potentially leading to DAD and alveolar edema, as described by Rockx et al. [29]. Epithelial desquamation was observed in around half of patients with DAD in our study (Table 2). In our study, the amount of infected type I pneumocytes was very similar, regardless of DAD status, raising further questions concerning the pathogenesis of DAD (Table 3 and Figure 3A(II),B(I),B(IV)).

Type II pneumocytes, involved in surfactant production and regeneration, can become infected and contribute to inflammation, dysfunctional surfactant quantities, and injury to the alveolar-capillary barrier, all of which can lead to DAD and edema [30]. This coincides with the high number of cells found in our study within alveolar space, mainly because of desquamation (Table 3).

Macrophages act as phagocytes, engulfing infected cells and debris, but can also become infected and contribute to inflammatory cytokine release [31]. We have observed a high number of infected macrophages in all patients, regardless of the presence of DAD. Studies on the role of macrophages in DAD associated with COVID-19 are ongoing and describe a complex picture with some key themes, implying a dual role of macrophages as both protectors and contributors to injury [32], potentially triggering the cytokine storm by releasing excess IL-6 and TNF-α from phagocyted desquamated cells, creating an ever-aggravating loop of cytokine release [33]. Infected macrophages may also be responsible for viral persistence due to phenotype shifts [34]. Our observation of a higher number of infected macrophages in fatal COVID-19 cases (Table 3 and Figure 3) aligns with hypotheses proposed in previous studies, suggesting a potential link between extensive macrophage infection and the triggering of a cytokine storm that contributed to severe disease and death [33,34]. Frisoni et al. [11] conducted an immunohistochemical quantification on autopsies and reported the evidence of increased pro-inflammatory cytokines (e.g., IL-1β, IL-6, IL-15, and TNF-α), as well as immune cell infiltration in the lungs of COVID-19 patients. The findings reported by Frisoni et al. [11] support our data, linking cytokine storms to lung injury in COVID-19. Additionally, our histopathological findings mirror the elevated pro-inflammatory cytokine levels observed in COVID-19 patients with ARDS [35]. Furthermore, Yin et al. [36] compared clinical and flow cytometry data that included IL-6, IL-8, and TNF-α, and found a similar link between elevated cytokine levels and ARDS, as well as decreased lymphocyte counts in severe COVID-19 cases.

Endothelial cells can become infected, leading to vascular injury, blood clots, and potentially impaired oxygen delivery [37]. Otifi and Adiga [38] showed that direct viral infection leads to endothelial barrier disruption, which creates a pro-inflammatory and pro-coagulative state. Endothelial cells can also be affected by an ongoing cytokine storm occurring in the alveoli [39]. Our observation of a higher number of infected endothelial cells in fatal COVID-19 cases with DAD sustains the hypothesis of a potential interplay between extensive viral infection in the alveoli and the cytokine storm, leading to direct endothelial dysfunction and microthrombosis, while simultaneously the endothelial cells are affected by the virus itself, as it was present to a lower degree in cases without DAD put forth in previous studies by Pannone et al. [37], Otifi et al. [38], and Xu et al. [39]. Understanding the specific roles and interactions of these diverse cell types is crucial for developing targeted therapies, either by inhibiting the amount of cytokine released by cellular targeting or the direct targeting of cytokines and inactivating inflammatory molecules such as IL-6, IL-1, IL-17, and TNF-α [10].

Analysis of the macrophages revealed a median nuclear/cytoplasmic (N/C) ratio broadly consistent with healthy lung cells, as documented in a previous study [40]. This finding suggests an absence of altered N/C ratio and visible cytopathic changes, which are alterations in cell morphology caused by viral infection (Table 3). Notably, the N/C ratio did not show any association with the presence of DAD, indicating a potential decoupling between these two measures. This finding raises questions regarding the genesis of DAD as macrophage activation syndrome in COVID-19, which is well known to cause acute respiratory distress syndrome (ARDS) [41]. Unlike other viral infections that cause distinct cytopathic changes in lung tissue, SARS-CoV-2 infection appeared to lack these hallmarks in our study. This absence of specific cellular damage makes routine autopsy diagnosis of SARS-CoV-2 pneumonia challenging without confirmation by IHC or PCR. In comparison, HSV pneumonia exhibits characteristic intranuclear inclusions, CMV pneumonitis demonstrates enlarged cells with prominent inclusions, and RSV infection is known for inducing fused multinucleated cells [42].

### 4.2. The Perspective of a Cytological Evaluation in a Clinical Setting

Although sputum PCR is an essential tool for diagnosing SARS-CoV-2, it has limitations in identifying specific infected cell types and the immune response to the virus. Unlike sputum, bronchoalveolar lavage (BAL) offers a direct and targeted sampling of the lower airways, allowing for the quantification and morphological assessment of infected cell populations, particularly macrophages and pneumocytes. Through quantitative immunohistochemical analysis of BAL, we could predict prognosis regarding the potential development of DAD. This information could be crucial during high-stress pandemics, enabling informed triage decisions regarding intensive care admission based on individual patient risk profiles. Serial BAL analyses with immunohistochemistry and cytology could provide valuable insights into a patient’s response to treatment and disease progression, aiding in personalized treatment plans and risk stratification. Epithelial desquamation was a frequently observed finding in 33.3% of cases, especially in patients with DAD; this desquamation would facilitate high-quality cytologic specimens (Table 2). In our study, we have found infected cells floating within the alveolar space, primarily macrophages and pneumocytes; however, we have noticed a heterogeneous distribution of the lesions and infected cells, which may lead to possible errors in the diagnosis of BAL. While this study adds to the growing body of evidence supporting the potential of BAL in COVID-19 diagnosis and management, it is essential to acknowledge the existing reports. Several researchers [43,44] have already demonstrated the feasibility and potential benefits of BAL.

Broncho-alveolar cytology and immunohistochemical staining of cytoblocks could offer valuable insights into the cellular dynamics of SARS-CoV-2 infection. These tools can enhance our understanding of the disease and its progression and potentially support more targeted treatment strategies.

### 4.3. Immunohistochemistry as a Method of Virus Detection

While RT-PCR remains the gold standard for SARS-CoV-2 diagnosis, immunohistochemistry has emerged as a valuable tool in the detection of SARS-CoV-2, showcasing notable efficiency than other alternative methods. Immunohistochemistry provides valuable spatial information, visualizing viral antigen localization within infected cells, which can aid in understanding the viral distribution and potential tissue preference, as previously reported by Pesti et al. [45,46]. However, IHC requires specialized expertise for its interpretation, potentially limiting its widespread application. Therefore, the efficiency of IHC for SARS-CoV-2 detection is context-dependent, balancing its advantages in turnaround time and tissue visualization with potential limitations in sensitivity and widespread availability. Integrating IHC with other diagnostic methods, such as RT-PCR, may offer a synergistic approach to optimizing patient management and understanding COVID-19 pathogenesis. In situ, hybridization techniques also could offer high sensitivity for virus detection and can be used easily alongside IHC as it can be performed on paraffinized tissue but has limited use for detection in other organs, as demonstrated by Massoth et al. [47]. Immunohistochemistry (IHC) is particularly effective in retrospective studies using paraffin-embedded tissue (FFPE) thanks to its unique combination of long-term tissue preservation, established compatibility, and flexibility in marker selection [48]. Paraffin-embedded tissues, stored for decades at room temperature maintain good morphological and antigenic integrity, enabling researchers to analyze past cases, compare data across time points, and understand disease progression, treatment efficacy, and long-term outcomes. The IHC protocols are specifically designed for FFPE, allowing for the sensitive detection of proteins, including viral and bacterial antigens, making it ideal for studying marker presence and distribution in archived tissues [49]. While potential antigen degradation over time and limited RNA/deoxyribonucleic acid (DNA) availability are limitations, IHC’s ability to leverage archived FFPE tissues makes it a powerful tool for retrospective studies, offering valuable insights into disease processes while advancing our medical understanding of various diseases.

### 4.4. Limitations of the Study

We acknowledge several limitations that warrant consideration. For patients who died outside the medical system, access to detailed clinical data was limited. This lack of complete medical history might hinder a comprehensive understanding of their health profile and its potential influence on the pulmonary pathology observed in the study. While this approach aimed to target relevant regions, it might miss potential viral presence in other lung sections, potentially underestimating the full extent of infection. Acknowledging these limitations underscores the need for future studies using lavage fluid and biopsy samples in different stages of the disease, comprehensive clinical data collection, and potentially single-cell RNA analyses to provide a more complete picture of COVID-19 lung pathology across diverse patient populations.

Our study contributes to the understanding of the severity of COVID-19 infection in specific cell types and its link to observed histopathological lesions. However, it also reveals areas where further knowledge is needed. We acknowledge that our current study has limitations in pinpointing a single cell type as the definitive trigger for DAD. This highlights the need for future investigations, employing more advanced molecular or imaging techniques such as flow-cytometry and enzyme-linked immunosorbent assay (ELISA). Such approaches could provide higher resolution analysis, allowing us to conclusively determine the specific cellular origins of DAD.

The complexities of COVID-19 continue to pose challenges, especially in young individuals, where mortality, even in the unvaccinated cohort, can have unclear origins. We acknowledge that in some cases the virus may not directly cause death, but rather by an intricate and not yet fully understood reaction triggered by its presence. Furthermore, our ability to definitively link specific comorbidities to increased fatality remains limited. While this study explored potential associations, statistically significant results were elusive, underscoring the need for further investigation.

## Figures and Tables

**Table 1:** Baseline characteristics of autopsy cases included in the study.

Characteristic	All *n* = 51	With DAD *n* = 24	Without DAD *n* = 27	Stat. (*p*-Value)
Age group				n.a. (0.945) *
<50	4 (7.8)	2 (8.3)	2 (7.4)	
50–59	7 (13.7)	3 (12.5)	4 (14.8)	
60–69	13 (25.5)	7 (29.2)	6 (22.2)	
≥70	27 (52.9)	12 (50)	15 (55.6)	
Sex				1.5 (0.226)
female	13 (25.5)	8 (33.3)	5 (18.5)	
male	38 (74.5)	16 (66.7)	22 (81.5)	
Weight status				n.a. (0.400)
underweight	9 (17.6)	3 (12.5)	6 (22.2)	
normal	13 (25.5)	8 (33.3)	5 (18.5)	
obesity	29 (56.9)	13 (54.2)	16 (59.3)	

Data are shown as no. (%); * Stat. is the χ^2^ statistics for categorical data, Fisher’s exact test for small samples; DAD = Diffuse alveolar damage; n.a. = not applicable.

**Table 2:** Microscopic features in lung tissue.

Feature	All *n* = 51	With DAD *n* = 24	Without DAD *n* = 27	Stat. (*p*-Value)
Alveolar edema	44 (86.3)	23 (95.8)	21 (77.8)	n.a. (0.061) *
Interstitial pneumonia	37 (72.5)	21 (87.5)	16 (59.3)	3.1 (0.024)
Microthrombi	25 (49.0)	11 (45.8)	14 (51.9)	0.2 (0.668)
Antrachotic pigment	20 (39.2)	6 (25.0)	14 (51.9)	3.8 (0.050)
Epithelial desquamation	17 (33.3)	13 (54.2)	4 (14.8)	8.9 (0.003)

Data are shown as no. (%); * Stat. is the χ^2^ statistics for categorical data, Fisher’s exact test for small samples; DAD = Diffuse alveolar damage; n.a. = not applicable.

**Table 3:** Morphometric and immunoreactivity characteristics of infected cells.

Characteristics	All *n* = 51	With DAD *n* = 24	Without DAD *n* = 27	Stat. (*p*-Value)
Type I pneumocytes				0.5 (0.653) ^#^
median [Q1 to Q3]	4 [0 to 11]	4 [1 to 11]	4 [0 to 10]	
{min to max}	{0 to 44}	{0 to 44}	{0 to 37}	
IRS type I Pn *				n.a. (0.633)
low	18 (35.3)	7 (29.2)	11 (40.7)	
moderate	28 (54.9)	14 (58.3)	14 (51.9)	
intense	5 (9.8)	3 (12.5)	2 (7.4)	
Macrophages				0.2 (0.836)
median [Q1 to Q3]	51 [23 to 108]	58 [22 to 129]	50 [25 to 95]	
{min to max}	{5 to 304}	{6 to 304}	{5 to 297}	
IRS Macrophage *				n.a. (0.773)
low	3 (5.9)	1 (4.2)	2 (7.4)	
moderate	45 (88.2)	22 (91.7)	23 (85.2)	
intense	3 (5.9)	1 (4.2)	2 (7.4)	
Endothelial cells				−0.5 (0.604)
median [Q1 to Q3]	2 [0 to 6]	5 [0 to 8]	1 [0 to 4]	
{min to max}	{0 to 40}	{0 to 24}	{0 to 40}	
IRS Endothelium *				n.a. (0.737)
absent	21 (41.2)	8 (33.3)	13 (48.1)	
low	4 (7.8)	2 (8.3)	2 (7.4)	
moderate	21 (41.2)	11 (45.8)	10 (37.0)	
intense	5 (9.8)	3 (12.5)	2 (7.4)	
Nucleus				−0.3 (0.763)
median [Q1 to Q3]	7 [6 to 9]	7 [6 to 8]	9 [7 to 9]	
{min to max}	{4 to 13}	{4 to 11}	{5 to 13}	
N/C ratio				−1.7 (0.083)
median [Q1 to Q3]	0.32 [0.22 to 0.54]	0.29 [0.21 to 0.47]	0.35 [0.23 to 0.55]	
{min to max}	{0.07 to 2.54}	{0.07 to 1.5}	{0.09 to 2.54}	

Results are reported as median and IQR for non-normally distributed and ordinal data except for * when number (%) was reported; ^#^ Stat. is the Z value associated with the Mann–Whitey test; IRS = immunoreactive score; IRS type I Pn = immunoreactive score for type I pneumocytes; N/C = nucleus/cytoplasm ratio; DAD = Diffuse alveolar damage; n.a. = not applicable.

## Data Availability

The data reported in this study are part of an ongoing Ph.D. thesis, so they are not publicly available. However, the raw data associated with this study can be obtained upon reasonable request addressed to the first author.
